# Can patients with femoral neck fracture benefit from preoperative thromboprophylaxis? A prospective randomized controlled trial

**DOI:** 10.1097/MD.0000000000007604

**Published:** 2017-07-21

**Authors:** Qiangqiang Li, Bingyang Dai, Jiacheng Xu, Yao Yao, Kai Song, Haojun Zhang, Dongyang Chen, Qing Jiang

**Affiliations:** a Department of Sports Medicine and Adult Reconstructive Surgery, Nanjing Drum Tower Hospital, Clinical College of Nanjing Medical University; b Laboratory for Bone and Joint Diseases, Model Animal Research Center, Nanjing University; c Department of Sports Medicine and Adult Reconstructive Surgery, Nanjing Drum Tower Hospital Affiliated with the Medical School of Nanjing University, Nanjing, Jiangsu; d Department of Orthopedics, The first Affiliated Hospital of Nantong University, Nantong; e NingBo No.6 Hospital, Ningbo, P.R. China.

**Keywords:** deep vein thrombosis, femoral neck fracture, rivaroxaban

## Abstract

**Background:**

The effectiveness of preoperative thromboprophylaxis remains obscure in patients with femoral neck fracture. The purpose of the current study was to investigate whether these patients benefit from preoperative thromboprophylaxis.

**Methods:**

In this prospective, randomized controlled trial, a total of 80 patients with femoral neck fracture were randomly assigned to receive either rivaroxaban or conservative treatment before surgery. For all patients, color Doppler ultrasound of both lower extremities was performed immediately after admission. The primary efficacy outcome was venous thromboembolism (VTE) defined as deep vein thrombosis (DVT) or pulmonary embolism (PE). The primary safety outcome was major bleeding.

**Result:**

Compared with conservative treatment, rivaroxaban could significantly reduce the incidence of DVT from 19.5% (8/41) to 2.6% (1/39) (*P* = .016). Preoperatively, there were a total of 9 occurrences of DVT including 8 DVT in the conservative treatment group and 1 in the oral rivaroxaban group. All cases of DVT were asymptomatic, with 8 of them diagnosed as isolated muscular calf vein thromboses. There were no differences between the 2 groups in terms of the overall incidence of major bleeding.

**Conclusion:**

Thromboprophylaxis with rivaroxaban prior to surgery can effectively reduce the risk of preoperative DVT for patients with femoral neck fracture without increasing the risk of bleeding.

## 1 Introduction

Patients with hip fracture are at a high risk of postoperative venous thromboembolism (VTE) because of their advanced age and frailty.^[1,2]^ This risk has been well documented by previous studies,^[3–6]^ and guidelines have recommended mechanical and pharmacological prophylaxis for patients undergoing surgery for hip fracture.^[7]^ To our knowledge, however, few investigators have concentrated on the preoperative prevalence of thrombosis in these high-risk patients, although the occurrence of preoperative deep vein thrombosis (DVT) was reported to occur in 9% to 62% of patients, even when they were receiving prophylaxis.^[8–10]^ Moreover, the inevitable delay of surgical intervention due to transfer from primary care facilities could add to the risk of preoperative DVT. Despite the high preoperative incidence of DVT in these patients, it remains controversial whether patients with femoral neck fracture can benefit from thromboprophylaxis before surgery. In Europe, anticoagulation therapy was usually initiated 12 hours before surgery^[11]^ to optimize antithrombotic effectiveness,^[12–15]^ because clinicians found that DVT typically developed perioperatively. However, Perka^[16]^ and Liu et al^[17]^ demonstrated that the effectiveness of preoperative initiation of anticoagulation therapy was comparable to that of postoperative anticaogulation therapy. In addition, opponents of preoperative anticoagulation therapy stated that it could add to the risk of postoperative intraspinal hematoma^[18]^ and lead to longer delays of surgery.^[19]^ Such discrepancy in clinical practice has led to a need to determine the optimal timing for initiating anticoagulation therapy, which may facilitate the prophylaxis of DVT in patients with femoral neck fracture.

Rivaroxaban, a new and selective factor Xa inhibitor, has been reported to reduce the risk of VTE^[20]^ without a significant increase in major bleeding rates^[21]^ and is more effective than enoxaparin.^[20–23]^ But there is still a lack of evidence for rivaroxaban's use, particularly regarding its efficacy and safety in patients with femoral neck fracture. Herein, the aim of the present randomized clinical trial was to evaluate the benefit-to-risk ratio of 10 mg once daily oral rivaroxaban compared with conservative treatment in patients with femoral neck fracture. Our primary hypothesis was that preoperative thromboprophylaxis with rivaroxaban reduces the risk of preoperative DVT for these patients compared with those who received conservative treatment. A secondary hypothesis was whether preoperative thromboprophylaxis with rivaroxaban increases the risk of bleeding.

## 2 Material and methods

### 2.1 Patients

Between November 2014 and August 2015, 109 consecutive patients with femoral neck fracture were finally recruited for this study. Patients aged at least 60 years and with a body mass index (BMI) ≤30 kg/m^2^ were considered for inclusion. All patients received a bilateral color Doppler ultrasound scan immediately after admission. Exclusion criteria were as follows: having DVT on admission; presenting with trauma affecting more than 1 organ system; having more than 10 days between the causative trauma and hospital admission; requiring long-term anticoagulant for a chronic comorbidity condition; undergoing any type of anticoagulant or fibrinolytic therapy or dextran therapy; and having severe organ insufficiency such as congestive heart failure, liver insufficiency, renal failure, malignancy or chronic obstructive pulmonary disease. Twenty-nine patients were not randomized in this study because, among other reasons, 5 patients had confirmed DVT on admission, and there was lack of consent for 6 patients; however, the 2 study groups did not differ significantly with respect to reasons for exclusions. Finally, a total of 80 patients met the selection criteria for randomization and completed the study. Baseline characteristics were measured for each patient, including age, sex, history of thromboembolic disease, any comorbidities, intraoperative blood loss, postsurgical wound drainage, duration of surgery, and length of hospital stay.

### 2.2 Randomization and masking

This was a prospective, randomized, comparative trial. Approval was obtained from our ethics committee. Eligible patients were randomly assigned to 2 groups according to a computer-generated random sequence. Patients were assigned to receive treatment with oral rivaroxaban, 10 mg once daily, or conservative treatment from admission to surgery. The first drug dose was given the night of admission if there was no clinically evident bleeding. Prophylaxis was continued until 1 day before the operation. The patients in the conservative treatment group were instructed to stay in bed and keep mobilization without receiving any thromboprophylaxis drugs. Postoperatively, all patients received rivaroxaban 6 to 8 hours after surgery. The administration of rivaroxaban was scheduled for 7 to 11 days. In case of confirmed VTE, the anticoagulation treatment was discontinued and conventional thrombolysis treatment was then performed. The end point of this study was the moment when the patients finished the treatment. Drains were removed when the drainage volume was less than 50 mL/24 h. Written informed consent was obtained from all individual participants before randomization.

### 2.3 Medications

In the oral rivaroxaban group, patients were treated with oral rivaroxaban, 10 mg once daily. In the conservative treatment group, patients did not receive prophylaxis before surgery. Throughout the study, the following medications that could affect the patients’ blood coagulation system were prohibited, including aspirin, non-steroidal anti-inflammatory drugs, low-molecular-weight heparin (LMWH), antiplatelet agents, anticoagulant, and thrombolytic agents. The use of a pressure pump was started on the first night after surgery.^[1]^

### 2.4 Outcome measures

The primary efficacy outcome was VTE defined as DVT detected by color Doppler ultrasound or pulmonary embolism (PE) recorded before discharge. Secondary efficacy outcomes were total, proximal, distal DVT, and symptomatic VTE.^[1]^ All the patients were examined for DVT by color Doppler ultrasound on admission, 1 day before the operation and 3 days after the operation or earlier if thrombosis was clinically suspected, which was diagnosed by 2 experienced doctors. Both of them were blinded to the randomization. DVT was diagnosed according to the Lensing group's criterion,^[24]^ and PE was diagnosed by computed tomography (CT) scan for pulmonary artery angiogram.

The primary safety outcome was major bleeding recorded before discharge, including fatal bleeding, retroperitoneal, intracranial, or intraspinal bleeding, bleeding that involved any other critical organ, bleeding associated with severe anemia and surgical bleeding ≥1200 mL.^[1]^ All the other bleeding events were classified as minor. Secondary safety outcomes were all deaths, any other bleeding events, transfusion requirements, thrombocytopenia, and any other adverse events.

### 2.5 Statistical analysis

Statistical analysis was performed using SPSS 19.0 system software (SPSS Inc., Chicago, IL). Numeric variables were compared by Student *t* test and categorical variables were compared by the chi-square test. Numeric data are shown as mean ± standard deviation, categorical data are shown as numbers with percentages, and continuous skewed data are shown as medians (ranges). A value of *P* < .05 was considered statistically significant.

## 3 Results

### 3.1 Study population

**Figure 1:**
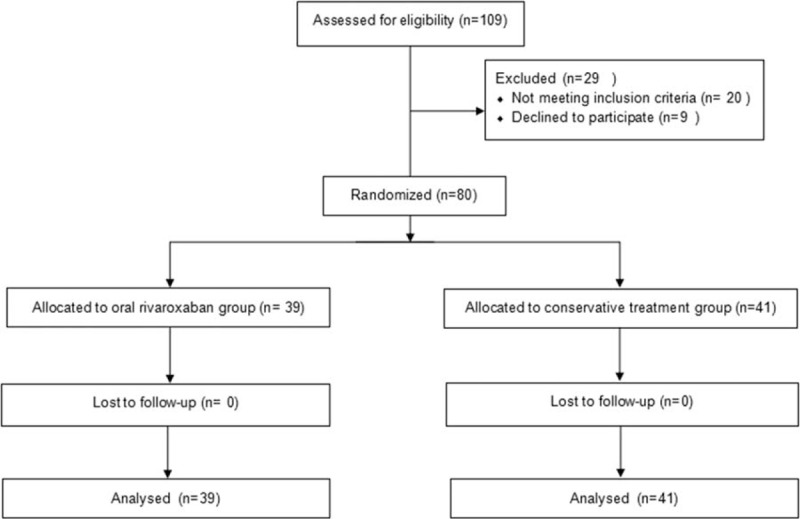
Flow diagram according to the consolidated standards of reporting trials (CONSORT) statement, showing recruitment, randomization, and patient flow in this study.

There were 52 women and 28 men patients with a mean age of 76.1 years (range, 60–94 years). Baseline characteristics did not differ significantly between the 2 groups (Table 1). Sixty-one patients underwent total hip arthroplasty and 19 patients underwent hemi-arthroplasty. The study groups did not differ in terms of type of surgery. Moreover, the 2 groups did not differ in the number of treatment days from admission to surgery. Details of the operation are summarized in Table 2. The mean duration between admission and surgery was 3.9 ± 1.9 days and 4.2 ± 2.2 days (*P* = .485) for the oral rivaroxaban group and the conservative treatment group, respectively. The mean duration of surgery was 87.0 ± 16.9 minutes and 84.1 ± 18.2 minutes (*P* = .467) for the oral rivaroxaban group and the conservative treatment group, respectively. The mean blood loss was estimated to be 178.6 ± 78.1 mL. Mean postsurgical wound drainage, collected from tubes placed according to surgical conventions, amounted to 219.9 ± 121.8 mL, and the mean time until drain removal was 2 days. The flowchart of the study is shown in Fig. 1.

**Table 1:**
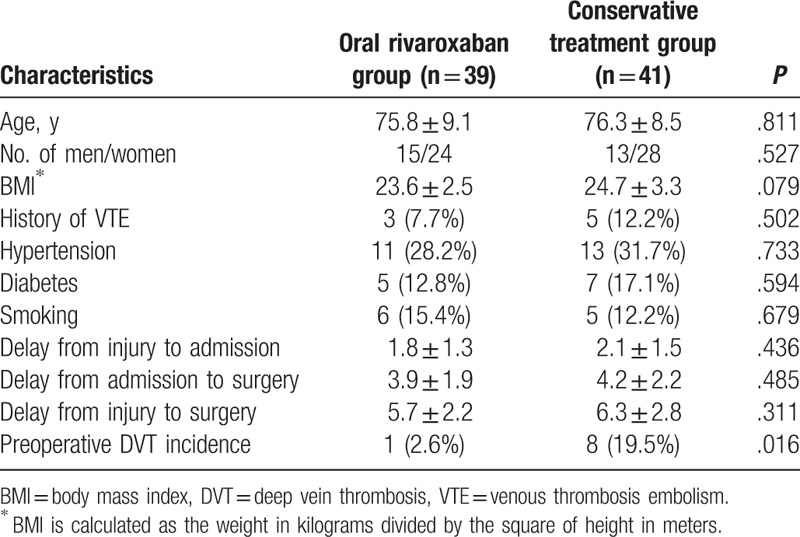
Baseline characteristics of the patients randomized.

**Table 2:**
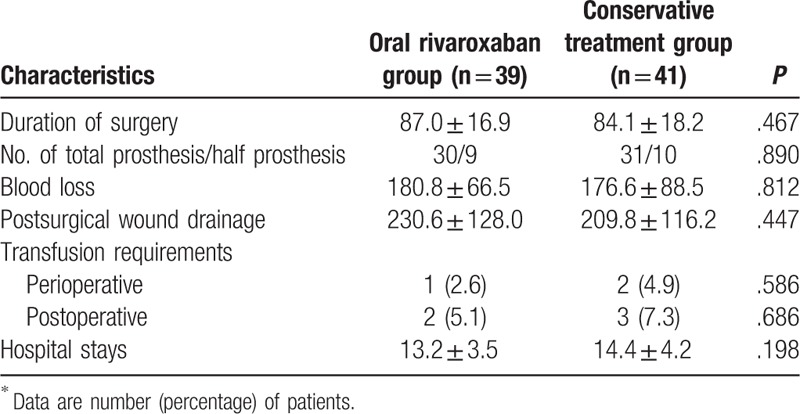
Surgical characteristics of the patients^∗^.

### 3.2 Incidence of VTE

All patients had both preoperative and postoperative color Doppler ultrasound screening. The incidence of preoperative DVT was significantly lower in the oral rivaroxaban group than in the conservative treatment group (2.6% vs 19.5%, *P* = .016). Before the operation, there were 9 incidences of DVT and 0 non-fatal PE. Eight incidences of DVT were detected in the conservative treatment group, and 1 incidence of DVT was detected in the oral rivaroxaban group (Table 3). All 9 cases of DVT were asymptomatic, and 7 of them were isolated muscular calf vein thromboses; in addition, 5 DVT were found in the ipsilateral extremity as the fracture, 3 patients had DVT in both extremities and 1 in the contralateral. All the 9 patients were successfully treated with thrombolysis and supportive measures. Moreover, following surgery, DVT was newly detected in 3 patients in the oral rivaroxaban group and in 4 patients in the conservative treatment group when the routine color Doppler ultrasound screening was evaluated 3 days postoperatively. In addition, 1 non-fatal PE occurred in the conservative treatment group, but there was no occurrence in the rivaroxaban-treated patients. Among the 7 incidences of postoperative DVT, 4 DVT were found in the ipsilateral extremity as the fracture, 2 in the contralateral, and there was 1 patient with DVT in both extremities. After including the incidences of DVT that occurred preoperatively, there was a significant reduction in the incidence of DVT with rivaroxaban compared with the conservative treatment (29.3% vs 10.3%, *P* = .034). If the preoperative DVT were excluded, there was no significant difference in the incidence of postoperative DVT between the 2 groups (7.7% vs 9.8%, *P* = .744).

**Table 3:**
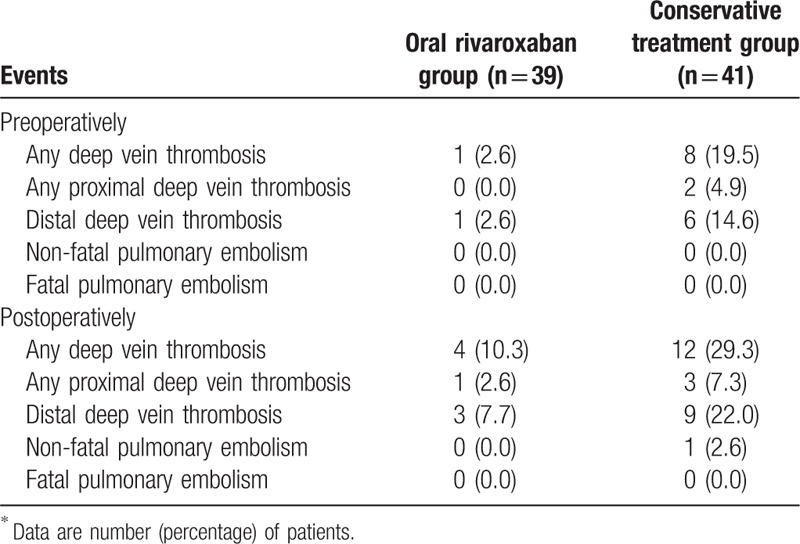
Incidence of venous thrombosis embolism events^∗^.

### 3.3 Safety outcomes

There was no fatal bleeding or bleeding in a critical organ in either treatment group. In the oral rivaroxaban group, 2 patients suffered from minor wound bleeding, indicated by wound ecchymosis and bleeding. In the conservative treatment group, 1 patient developed minor wound bleeding. No overt bleeding occurred throughout the study. The total primary safety outcome was therefore 2 minor bleeding episodes in oral revaroxaban group (5.1%), compared with 1 in the conservative treatment group (2.4%). The incidence of adverse events, including severe thrombocytopenia, and overall mortality did not differ between groups.

## 4 Discussion

Patients with femoral neck fracture are commonly at an increased risk of VTE.^[1]^ In a previous study, Roberts et al^[10]^ reported a 9% incidence of DVT in 176 patients with hip fracture prescribed with low-molecular-weight dextran. Smith et al^[25]^ reported an incidence of 11.9% of radiologically identifiable DVT in 101 patients who were given subcutaneous injections of heparin. Subsequently, several studies have investigated the incidence of thromboembolic disease in this patient group, the rate of which ranged from 10% to 62%.^[26,27]^ In this study, the incidence of DVT was 19.5% in the conservative treatment group, which was higher than that reported by Chan et al^[8]^ and by Cho et al.^[9]^ Of note however, Chan et al^[8]^ only recorded the DVT proximal to the popliteal vein, which could explain the low incidence in their study. Given the high incidence of preoperative DVT in patients without anticoagulation, it has been recommended that routine thromboprophylaxis should be initiated during the time between hospital admission and surgery.^[28]^ In addition, Hull et al^[11]^ found that the initiation timing of LMWH therapy significantly affected the effectiveness of DVT prophylaxis. However, some researchers have recommended against preoperative anticoagulation therapy because of the concern that it may increase the risk of bleeding complications such as the increase of intraoperative and postoperative blood loss^[15,16,29,30]^ and the rate of intraspinal hematoma.^[18]^ In addition, it has been reported that there was no difference between the preoperative and postoperative initiation of anticoagulation therapy regarding its efficacy and safety.^[11,14,31]^ The discrepancy in these studies reflects the need for a well-designed study that should comprehensively evaluate the effectiveness and the risk of initiating early prophylaxis.

For the first time, this study demonstrated that rivaroxaban can effectively prevent VTE in patients with femoral neck fracture when initiated immediately on hospital admission. This study shows that in this high-risk population, the incidence of preoperative DVT was markedly reduced when rivaroxaban was prescribed preoperatively at a dose of 10 mg per day. Previous studies have well documented the relationship between the timing of initiation of LMWH therapy and the effectiveness of DVT prophylaxis.^[11,32]^ However, some studies have observed limited effectiveness of anticoagulation therapy in preventing DVT.^[16,17]^ Liu et al^[17]^ retrospectively evaluated 222 patients with hip fracture, who were allocated to 2 groups receiving either preoperative or postoperative initiation of LMWH therapy. They concluded that preoperative anticoagulation therapy may not increase intraoperative and postoperative blood loss, and the risk of postoperative DVT or PE. We noted that in these studies, however, the authors identified DVT primarily based on clinical symptoms without performing routine venography or color Doppler ultrasound for patients, thereby reducing the accuracy of their outcome data. By contrast, in the current study, color Doppler ultrasound was performed for every single patient, which added to the accuracy of the diagnosis of early asymptomatic DVT.

As a complication of chemoprophylaxis, the incidence of bleeding was also of concern to surgeons when deciding on the initiation time of anticoagulation therapy.^[31,33]^ Some authors have observed a risk of bleeding and an increase of intraoperative and postoperative blood loss.^[15,16,29,30]^ In this study, preoperative anticoagulation therapy with rivaroxaban did not appear to increase the risk of minor and major bleeding events. Moreover, no significant differences were detected in terms of intraoperative blood loss and postoperative wound drainage in the 2 groups, which was similar to the results found by previous studies.^[16,17]^

In this study, 9 patients with preoperative DVT were treated without using the filter. None of them showed any sign of PE and the thrombosis resolved spontaneously later during their care. To our knowledge, retrievable inferior vena cava (IVC) filters were previously frequently used in patients with DVT undergoing orthopedic surgeries.^[25,34]^ However, a recent randomized clinical trial with a 6-month follow-up demonstrated that patients with acute VTE did not benefit from the use of a retrievable IVC filter.^[35]^ Thus, for patients with preoperative DVT who are at the risk of an intraoperative pulmonary embolism, further studies are needed to investigate whether the IVC filter should be applied.

Despite the fact that our data were derived from a randomized controlled trial, several limitations still existed in our study. First, the patient sample of this study was relatively small, which could lead to insufficient statistical power to detect small difference in clinical outcomes. Second, the average time period before surgery in both groups was between 3 and 4 days, which is longer than that of most US hospitals, and this prolonged immobility probably predisposed these patients to developing DVT. However, some necessary delays of surgery are common in tertiary care facilities caused by transferring from community hospitals and by preoperative assessment and optimization. Third, only early postoperative incidences of DVT were tested by using color Doppler ultrasound, which may lead to missing patients with DVT after discharge. However, it has been reported that more than 85% of documented DVTs occur within 1 day after surgery.^[36]^ Moreover, once patients were diagnosed with negative DVT following early color Doppler ultrasound in our study, they would subsequently receive an aggressive rehabilitation program, which might have reduced the risk of DVT substantially.

## 5 Conclusions

Thromboprophylaxis with rivaroxaban prior to surgery can effectively reduce the risk of DVT for patients with femoral neck fracture without increasing the bleeding rates. We recommend routine thromboprophylaxis with rivaroxaban on the first day of the patients’ admission.
